# Volatile Organic Compound Profile Fingerprints Using DART–MS Shows Species-Specific Patterns in *Fusarium* Mycotoxin Producing Fungi

**DOI:** 10.3390/jof8010003

**Published:** 2021-12-21

**Authors:** Mark Busman, Ethan Roberts, Robert H. Proctor, Chris M. Maragos

**Affiliations:** USDA, Agricultural Research Service, National Center for Agricultural Utilization Research, Mycotoxin Prevention and Applied Microbiology Research Unit, 1815 N. University, Peoria, IL 61604, USA; ethan.roberts@usda.gov (E.R.); robert.proctor@usda.gov (R.H.P.); chris.maragos@usda.gov (C.M.M.)

**Keywords:** volatile organic compounds, *Fusarium*, DART–MS, mycotoxins

## Abstract

Fungal volatile organic compounds (VOCs) are low-molecular weight fungal metabolites that have high vapor pressure at ambient temperatures and can function as airborne signals. Here, we report a VOC study of several different species of *Fusarium*. Direct analysis in real time mass spectrometry (DART–MS) was applied for non-invasive VOC fingerprinting of *Fusarium* isolates growing under standardized conditions. A large number of ions were detected from the headspaces of the *Fusarium* species sampled here. Ions were detected with distinctively high concentrations in some species. While there were few VOCs produced by only one species, the relative concentrations of VOCs differed between species. The methodology has potential for convenient detection and identification of *Fusarium* contamination in agricultural commodities.

## 1. Introduction

Fungal species belonging to the genus *Fusarium* live on a wide range of substrates. However, they are widely known to infest plants [1] and are the causal organisms behind important crop disease such as Fusarium head blight in wheat [2] and *Fusarium* ear rot in maize [3]. Further, *Fusarium* species are responsible for the mycotoxin contamination of crops [4,5]. Mycotoxins are secondary metabolites of the infesting fungi produced by a variety of metabolic pathways including those based upon terpenoid and polyketide backbones. In recent decades, the phylogeny of *Fusarium* has been resolved based on a multi-genome phylogeny [6]. Twenty-three species complexes are now recognized in *Fusarium* [7], including the *Fusarium sambucinum*, *F. incarnatum-equiseti*, *F. fujikuroi*, *F. tricinctum*, and *F. solani* species complexes [6]. Methods for rapid and convenient identification of mycotoxin producing fungi are helpful for grain producers in the management of growing crops while minimizing mycotoxin contamination [8].

Fungal volatile organic compounds (VOCs) are widely produced by fungi and can facilitate the information exchange between fungi and their environment [9,10]. VOCs might increase the competitive advantage of specific fungi or stimulate mutualistic interactions, enabling the colonization of habitats, depending on nutrient availability and other pre-colonization conditions [11]. Fungi produce different chemical classes of VOCs including alcohols, aldehydes, ketones, esters, and sesquiterpenes [4]. This variety suggests a potential for using the identity of the VOCs profiles as a signature that will allow for detection and identification of growing fungi. However, a knowledge gap exists regarding species-specific production of fungal VOCs [11] in a variety of fungal species. Presently, systematic VOC profile investigations based on multiple species within *Fusarium* are particularly lacking. There are experimental difficulties in studying the source and function of VOCs produced under natural conditions in developing plant and stored grain [3]. Development of techniques for examination of VOC production of individual species in pure culture on standard substrates is a useful starting point for the examination of VOC production under conditions more relevant to understanding plant disease [9]. Utilization of VOC analysis could aid identification of unique VOC signatures to enable detection and identification of specific fungal species.

Analytical techniques are available for both qualitative and quantitative analysis of VOCs [12,13]. Mass spectrometry (MS) has been utilized for the specific and sensitive analysis of VOCs [9]. VOC analyses have traditionally consisted of two steps: the first step is volatile collection, usually on some sort of absorbent such as activated charcoal, porous polymer adsorbent, or solid phase micro-extraction (SPME) fibers and is reliant on chemical adsorption / absorption and desorption. The second step is chemical analysis typically performed by MS. The SPME–GC–MS interface to electron-impact ionization (EI)–MS is the basis for much of the characterization VOC production by *Fusarium* [14]. The characteristics of SPME trapping, GC separation, and the EI source bias the VOC characterization towards certain types of VOCs [9].

Alternative MS techniques have been utilized to address VOCs that are not well analyzed by EI–MS techniques. Proton transfer reaction (PTR) MS is a technique that complements GC–MS-based methods [11]. PTR–MS is based on an alternative mode of ionization of the VOCs that allows for the analysis of VOCs. The PTR source utilizes medium vacuum source conditions (~2 mbar) to transfer a proton to a neutral VOC analyte molecule in order to generate a positively charged, protonated ion [10]. VOCs with a proton affinity higher than water can be ionized, mass analyzed, and detected [15].

Other related techniques based upon a proton transfer ionization mechanism for ionization have been utilized for the analysis of fungal VOCs. VOCs have been directly introduced into atmospheric pressure chemical ionization (APCI) MS sources [16]. APCI–MS has been used for several fungal studies [17]. Selected ion flow tube (SIFT)–MS has been utilized for the evaluation of VOCs from *Ascocoryne sarcoides* and *Aspergillus fumigatus* [18,19]. Secondary electrospray ionization (SESI) has been used to analyze the VOCs from baker’s yeast [20].

Direct analysis in real time mass spectrometry (DART–MS) is a newer ambient ionization technique that is widely utilized in food safety, forensic and industrial chemistry applications [21]. DART–MS has been shown to provide the basis for convenient, sensitive analysis of fungal toxins from a variety of matrices [22]. Normally, the DART–MS instrument is operated in a manner that allows a near simultaneous volatilization and ionization of analyte [21]. However, the configuration of DART–MS instrumentation allows adaptation to a wide variety of sample introduction geometries. Recently, applications have been reported utilizing remote volatilization of analyte followed by ionization in the source region of the MS. This configuration of DART–MS has been used to sample materials that are related to food taste and aroma [23,24].

In the present study, we used DART–MS to analyze the VOC profiles of several Fusarium species. The DART–MS instrument was configured to dynamically sample the VOC profile from the headspace of *Fusarium* growing on cracked maize substrate. The format of the DART–MS experimental apparatus allowed analysis of VOCs directly from the headspace of fungi, without the necessity of a trapping or chromatography step in the method. Goals of this work were to determine (1) whether VOC profiles could be sampled and analyzed by DART–MS, (2) whether DART–MS VOC profiles differed between *Fusarium* species, and (3) whether information from DART–MS VOC profiles could be used for species differentiation.

## 2. Materials and Methods

### 2.1. Fungal Isolates

*Fusarium* isolates and the *Aspergillus flavus* isolate used in this study (Table 1) were obtained as glycerol suspensions from the NRRL culture collection (nrrl.ncaur.usda.gov) at the National Center for Agricultural Utilization Research (Peoria, IL, USA). Glycerol suspensions (3 µL) were spotted on V8 juice agar medium in a 10 cm Petri plate, and grown in the dark for 3 days at 25 °C before use for inoculation of cracked maize kernel medium.

### 2.2. Cultivation of Fusarium Isolates for VOC Profile Analysis

VOC profiles were collected from the headspace of individual fungal isolates grown on 50 g of sterile cracked maize kernel substrate enclosed in a 250 mL screw cap glass media bottle. Prior to inoculation, cracked maize was prepared by wetting the substrate with 20 mL of water and autoclaving for 30 min. The sterile substrate was further wetted with 20 mL of sterile water and inoculated with a 3 mm square plug of fungal mycelium grown on V8 juice agar for 7 days. Once the cracked maize was inoculated, the bottle was vigorously shaken to distribute the fungal hyphae. To allow some air exchange, the culture bottle lids were loosened by 1/8th of a turn and incubated at 25 °C for 28 days in the dark. As controls, bottles containing sterile cracked maize substrate only (without fungal inoculum) were incubated at the same conditions and included in the VOC profile analysis. The VOC profiles were collected from the headspace of 3 independent bottles for each isolate by DART–MS on day 7, 14, 21, and 28 post incubation. The entire inoculation experiment was conducted twice. So, evaluations of VOCs reflect the examination of 6 bottles, 3 bottles from each repetition of the inoculation experiment.

### 2.3. DART–MS Apparatus

The DART ionization source was attached to an Exactive Classic high-resolution MS (Thermo Fisher, Milford, MA, USA). The Exactive Classic MS was controlled by the Tune program supplied by the MS vendor with Xcalibur version 3.1.1. Ions from the DART source were drawn into the MS instrument through the ion inlet tube heated to 325 °C. For experiments targeted at structure elucidation of observed *Fusarium* VOCs, the DART ionization source was attached to a QExactive high-resolution tandem MS. The QExactive MS was controlled by the Tune program supplied by the MS vendor.

**Figure 1:**
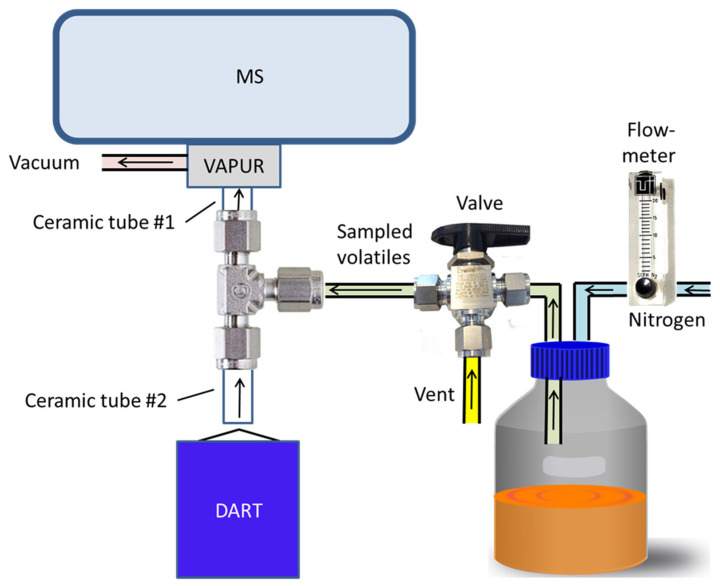
Cartoon representation of the modified DART–MS instrument constructed to allow convenient sampling of culture bottles.

The DART–MS instrument was configured for the sampling of the headspace above cultures in glass culture bottles as shown in Figure 1. The DART ionization source was a SVP–DART ion source (IonSense, Inc., Saugus, MA, USA). DART ionization was initiated by the SVP–DART ion source through a flow of helium that was heated to 300 °C. Control of the DART source was made through the DART–OS program from IonSense. Interface of the DART source to the MS was made with the VAPUR inlet fitted to the inlet region of the MS. Vacuum to the VAPUR inlet was supplied by a Vacuubrand ME2NT diaphragm pump (Vacuubrand Inc., Essex, CT, USA). Simultaneous inlet of ions from the DART source and vapor from the bottles containing fungi growing on cracked maize media was made through a 0.25-inch stainless steel Tee (Model SS-400-3, Swagelok Co., Solon, OH, USA) placed in front of the VAPUR inlet. Ceramic tube #1 had a 0.25 inch outside diameter (OD) × 4.75 mm inside diameter (ID) × 4.2 cm length and was obtained from IonSense. Ceramic tube #2 was 0.25 inch OD × 4.45 cm long, had two 0.062 mm ID passages and was obtained from Scientific Instrument Services (Palmer, MA, USA). Ceramic tubes were secured to the stainless-steel Tee by graphite ferrules (Scientific Instrument Services). Nitrogen gas flow to the culture bottle was conducted through 0.125 inch OD × 0.0625 inch ID polytetrafluoroethylene (PTFE) tubing and controlled to a flow rate of 3.5 L/min with a Model 132460-44 Cole-Parmer gas flowmeter. Gas flow from the headspace of the culture bottle to the DART source was conducted through 0.25 inch OD × 0.19 inch ID polytetrafluoroethylene (PTFE) tubing and controlled with a Swagelok Whitey 3-way 0.25 inch B-43 × S4 ball valve. Connection to the culture bottle was made through a 5-port GL45 bottle cap (2270–0013, Fisher Scientific). Nitrogen flow for the experiment was obtained from the boil-off from a liquid nitrogen dewar.

### 2.4. DART–MS Analysis

Measurements of fungal VOC profiles were performed by sampling of the headspace of pure cultures into the above-described DART–MS apparatus. The sample headspace was withdrawn through the DART–MS inlet with an analysis time of 3 min/sample. Pure nitrogen was flushed continuously through the bottle to prevent pressure drop. For each bottle, sampling time from the culture was bracketed by a 1 min collection of room air for DART–MS analysis both prior to and following sampling from the bottle. The Exactive Classic MS was operated in positive mode with a scan range of 75–1000 Da in profile mode. MS resolution for the experiments was set to “Enhanced Resolution” (25,000), scan rate 4 Hz, and the automatic gain control was set to “Ultimate mass accuracy (5 × 10^5^ ions)”. The QExactive MS was operated in positive mode with a scan range of 75–1000 Da in profile mode. MS resolution for the experiments was set to 70,000, scan rate 3.7 Hz, and the automatic gain control was set to “Ultimate mass accuracy (5 × 10^5^ ions)”. Collision energy for all CID experiments was 30 V.

### 2.5. Data Analysis

Data were analyzed using the QualBrowser component of the Xcalibur MS program supplied by the MS vendor. Empirical formulae for distinctive ions were suggested by use of the Compound Discover 3.2 program supplied by the MS vendor.

## 3. Results

### 3.1. DART–MS Sampling of the Headspace above Fungal Cultures Yielded Intense Ions

Under the culture conditions employed, visible fungal growth was already observed by day 7 and continued through the 28-day experiment. This was true for the *Fusarium* species and the *A. flavus* isolate used for comparison.

**Figure 2:**
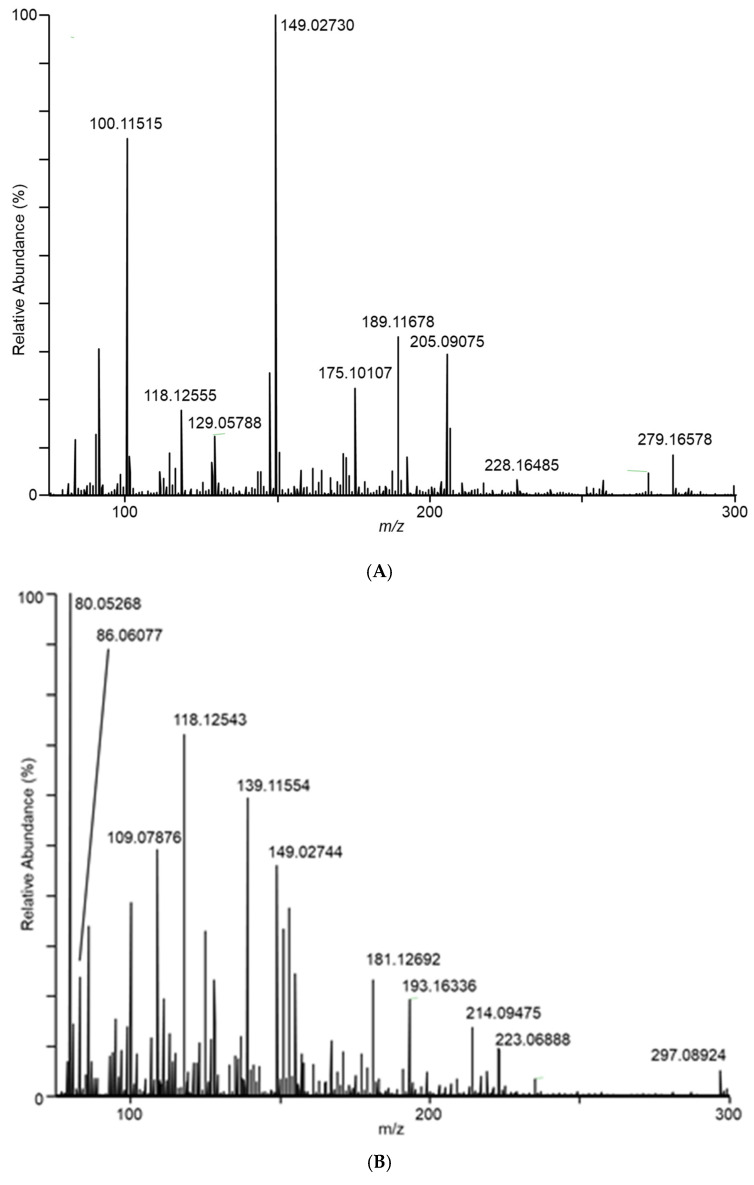

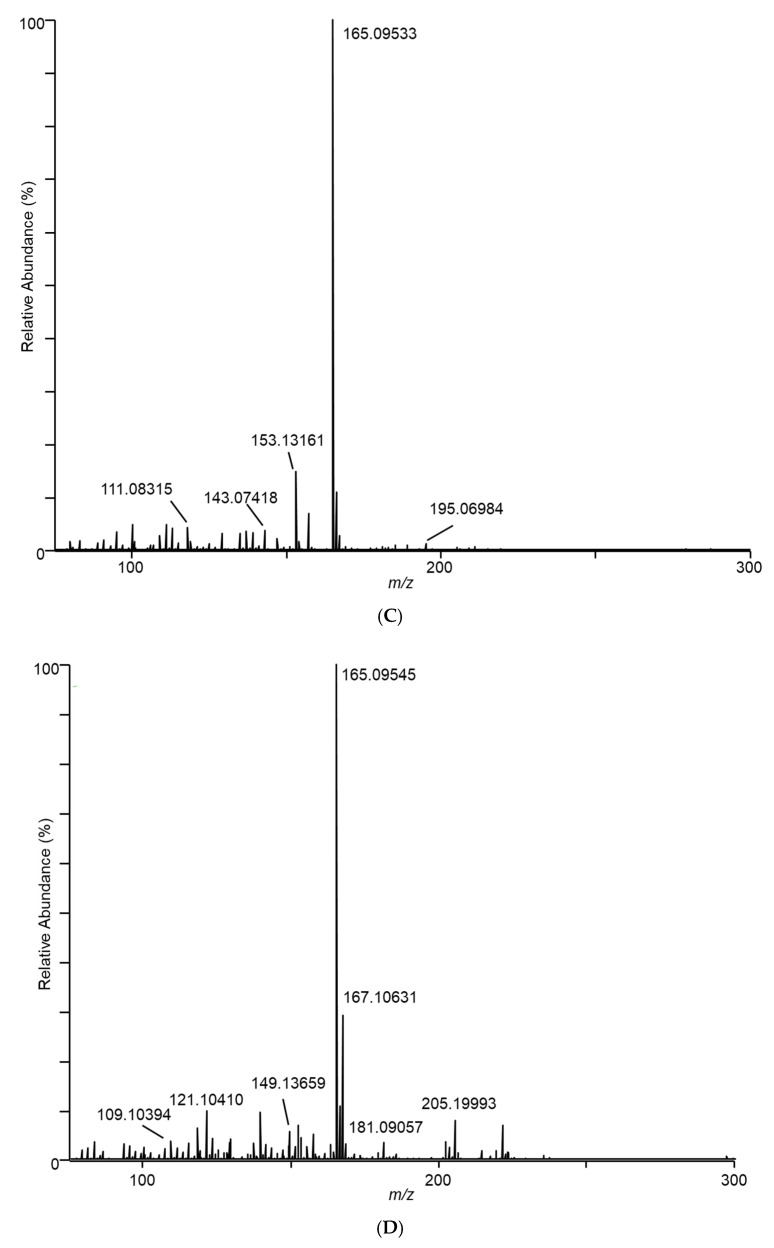

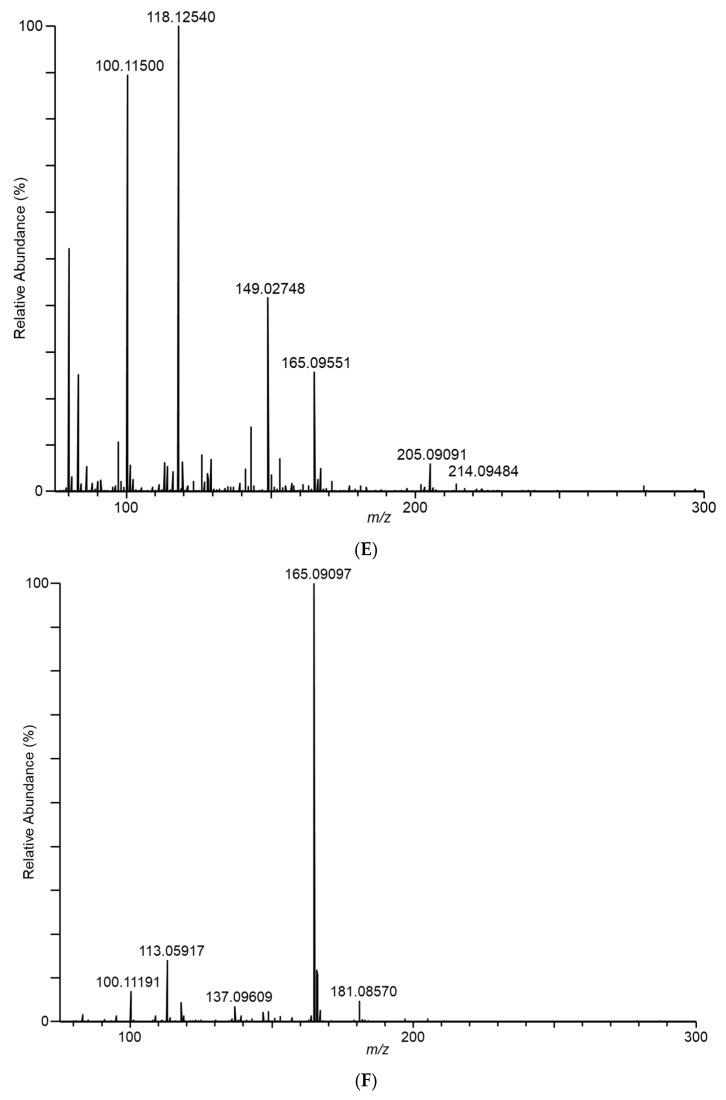

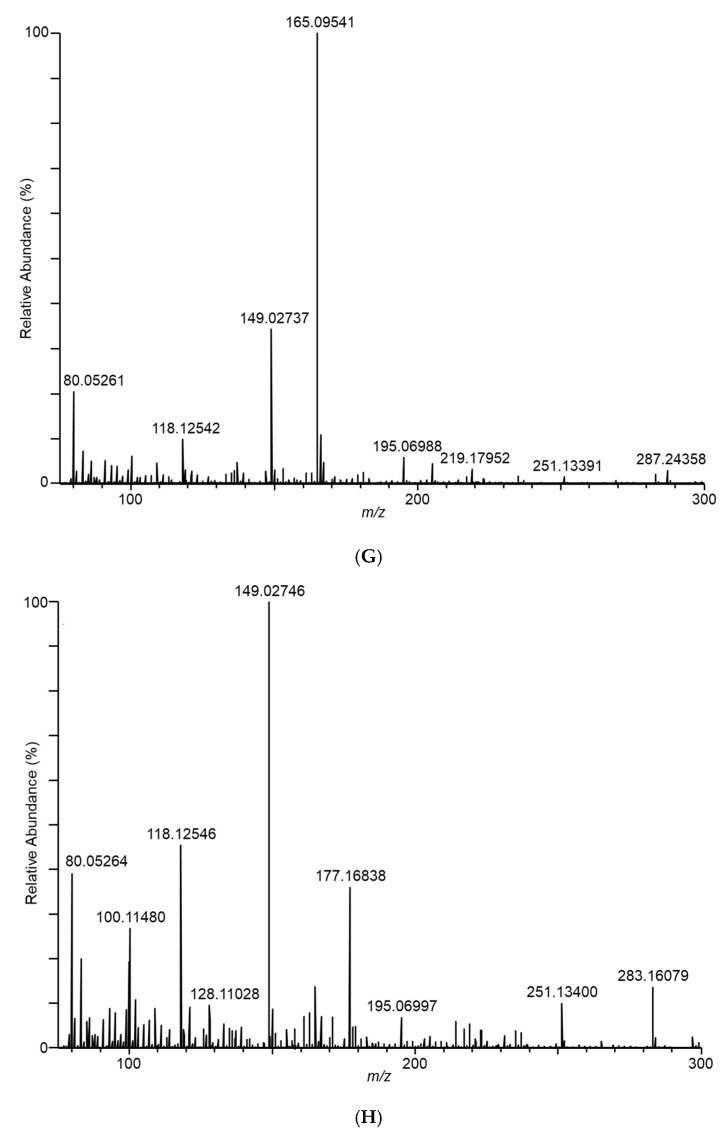
Spectra for (**A**) room air during conduct of the study, (**B**) control cracked maize, (**C**) *F. verticillioides* on cracked maize, (**D**) *F. scirpi* on cracked maize, (**E**) *F. avenaceum* on cracked maize, (**F**) *F. graminearum* on cracked maize, (**G**) *F. virguliforme* on cracked maize, and (**H**) *A. flavus* on cracked maize. Spectra represent averages of the 3 min acquisition period for each sample.

Differences in the spectra from the different fungal samples were apparent even at the first sampling, at day 7. As an example of the DART–MS sampling of the headspace, Figure 2 shows DART–MS spectra for the day 14 sampling of (A) room air, (B) control cracked maize, (C) *F. verticillioides* on cracked maize, and (D) *F. scirpi* on cracked maize. Spectra B, C, and D reflect the results of background subtraction to reduce the background ions inherent to the DART experiment. For the background subtraction utility in the QualBrowser program, 30 s periods before and after sampling were subtracted from the 3 min sampling period. During these two 30 s periods, the DART spectra collected were from the flow of room air drawn in as a result of the position of the sampling valve. The spectra illustrate the effective interface of DART–MS for the headspace sampling and provide visual confirmation of the differences between the volatile species accessible to the DART–MS sampling experiment.

In Figure 2C, ions *m/z* 165 and 153 dominate in the sampled headspace for *F. verticillioides* on cracked maize. Similarly, in Figure 2D, ions *m/z* 165 and 167 dominate in the sampled headspace for *F. scirpi*. The *m/z* 167 peak is not observed at substantial levels in the headspace from other fungi. The distinctive *m/z* 167 peak (167.10631) is readily resolved from the isotopic peak expected to result (167.10225) from two 13C atoms being incorporated into the molecule responsible for the large *m/z* 165 (165.09545) peak (data not shown). Higher levels of the *m/z* 165 peak are observed in the headspace from *F. graminearum*, *F. verticillioides*, and *F. scirpi*.

**Figure 3:**
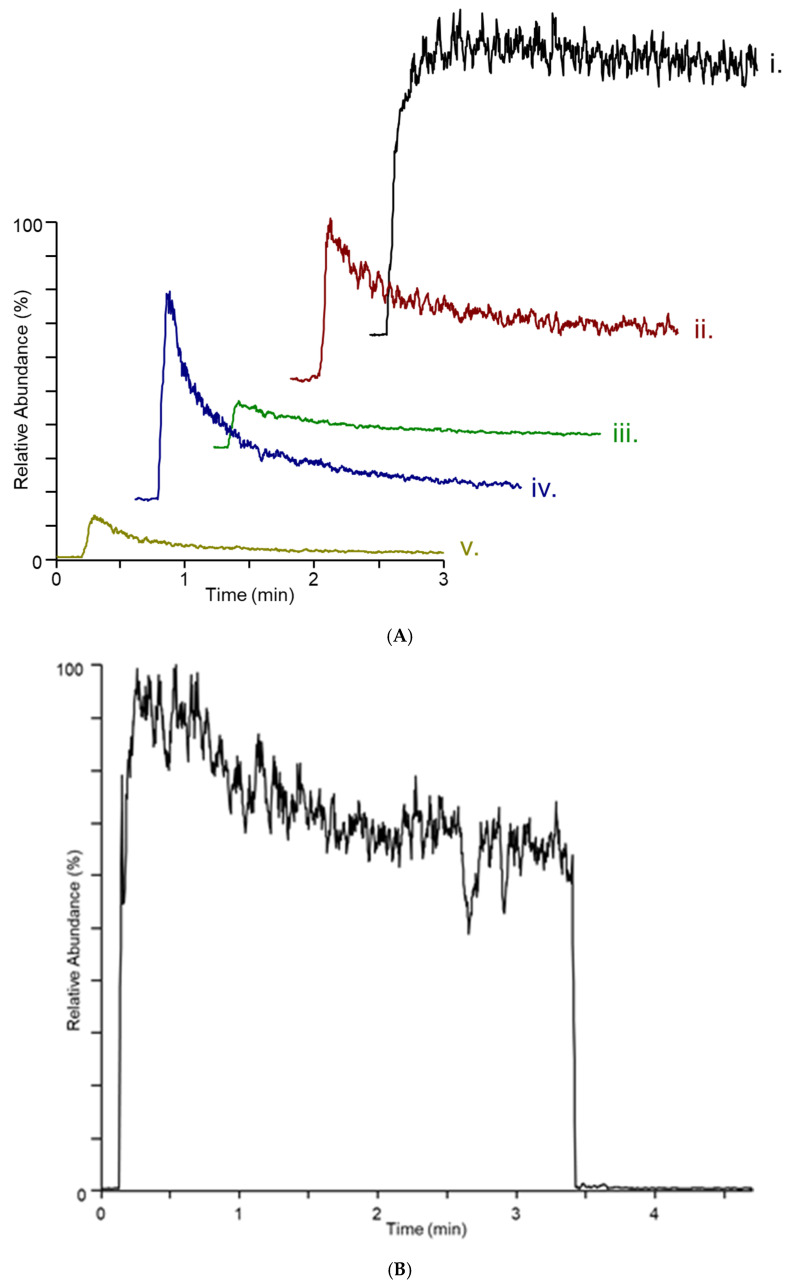
Plots of extracted ion intensity versus time for (**A**) *m/z* 165 from (i.) *F. verticillioides,* (ii.) *F. virguliforme*, (iii.) *F. graminearum* (iv.) *F. scirpi*, and (v.) *F. avenaceum* and (**B**) *m/z* 195 from *F. verticillioides* on cracked maize. The full scale for count intensity for plot (**A**) is 1.26 × 10^7^ and for plot (**B**) is 1.88 × 10^5^.

While the DART–MS experiment does analyze the headspace area above the cracked maize substrate, as the headspace vapor is swept from the culture bottle, differences in the dynamics of the sampling, ionization, and mass analysis are observed. Figure 3A,B show the response of the MS for the *m/z* 165 and 195 ions from a 14-day culture of *F. verticillioides*. The *m/z* 165 trace shows a rapid increase upon initiation of sampling followed by a modest decline, while the *m/z* 195 trace indicates a more modest increase at the beginning of the sampling, followed by a slower decrease until the end of sampling. It is not clear whether these dynamics are a consequence of the release of VOCs from the fungal colony on the substrate or whether they are an artefact of the instrument geometry.

### 3.2. Inspection of Spectra from Control Cracked Maize Bottles Yielded a Distinctive Ion for the Cracked Maize Substrate

Using DART–MS, mass peaks were detected after filtering across all cracked maize samples in the test. Many of the high intensity peaks in the raw spectra from the DART–MS are inherent to the DART process. This is especially true at low *m/z*. Only one distinctive ion (*m/z* 86.06077) was noted for cracked maize under the culture conditions employed. The ion was present at substantial levels with all inoculants and with the control cracked maize samples. The level of the diagnostic ion for maize substrate appeared to be stable, regardless of experiment time and degree of fungal growth.

### 3.3. Across the Fusarium Species Sampled, There Were a Few Common Volatile Compounds Detected

**Figure 4:**
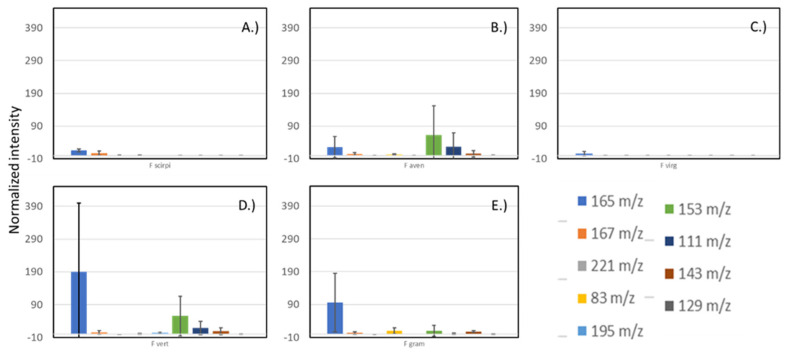
Intensities of selected ions from week 2 of sampling from cultures of (**A**) *F. scirpi*, (**B**) *F. avenaceum*, (**C**) *F. virguliforme*, (**D**) *F. verticillioides*, and (**E**) *F. graminearum*. Intensities are normalized relative to the diagnostic ion for cracked maize (*m/z* 86). Intensities shown are averages of measurements conducted, and error bars indicate calculated standard deviations for the six measurements.

For example, the *m/z* 165 ion was seen in all the *Fusarium* cultures but was not observed in the *Aspergillus flavus* cultures (data not shown). In fact, none of the selected ions for the *Fusarium* species were observed from the A. flavus cultures. This indicates that detected ions can reflect volatiles unique to the fungal species examined. While several mass peaks were detected in samples from multiple *Fusarium* species, there were some peaks found solely at significant levels in one species. Consequently, we did note species-specific mass peaks that could potentially be used as indicators for species identification. Figure 4 shows presence of ions visually selected as indications of fungal growth. Table 2 lists some distinctive ions visually selected for discrimination between inoculums. Ions were visually selected based upon uniqueness to the species instead of merely selecting them based upon the highest MS intensity VOC detected. No suitable diagnostic ions were found for *F. virguliforme*, and only one distinctive ion was identified for *F.* *avenaceum*.

**Figure 5:**
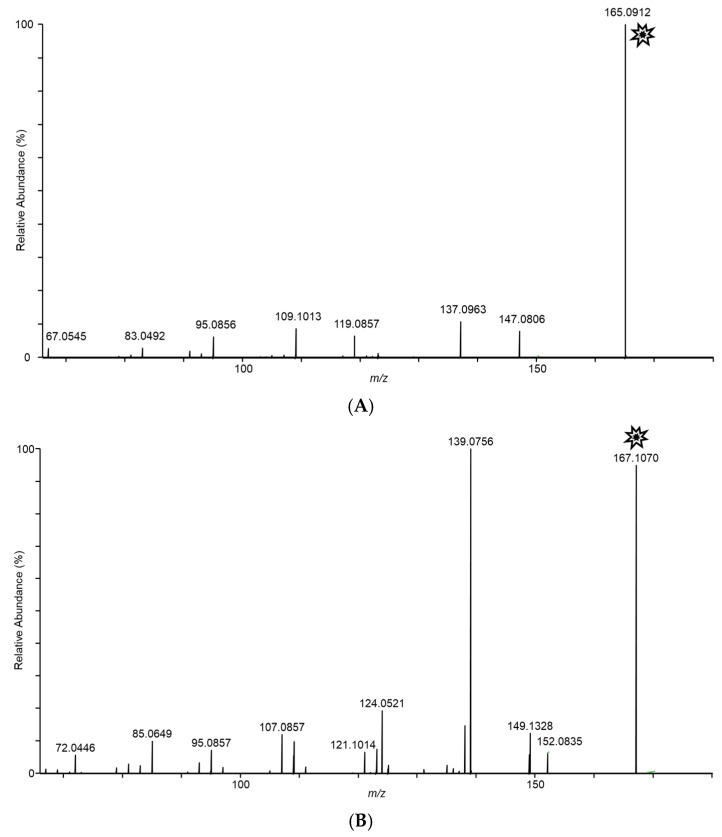

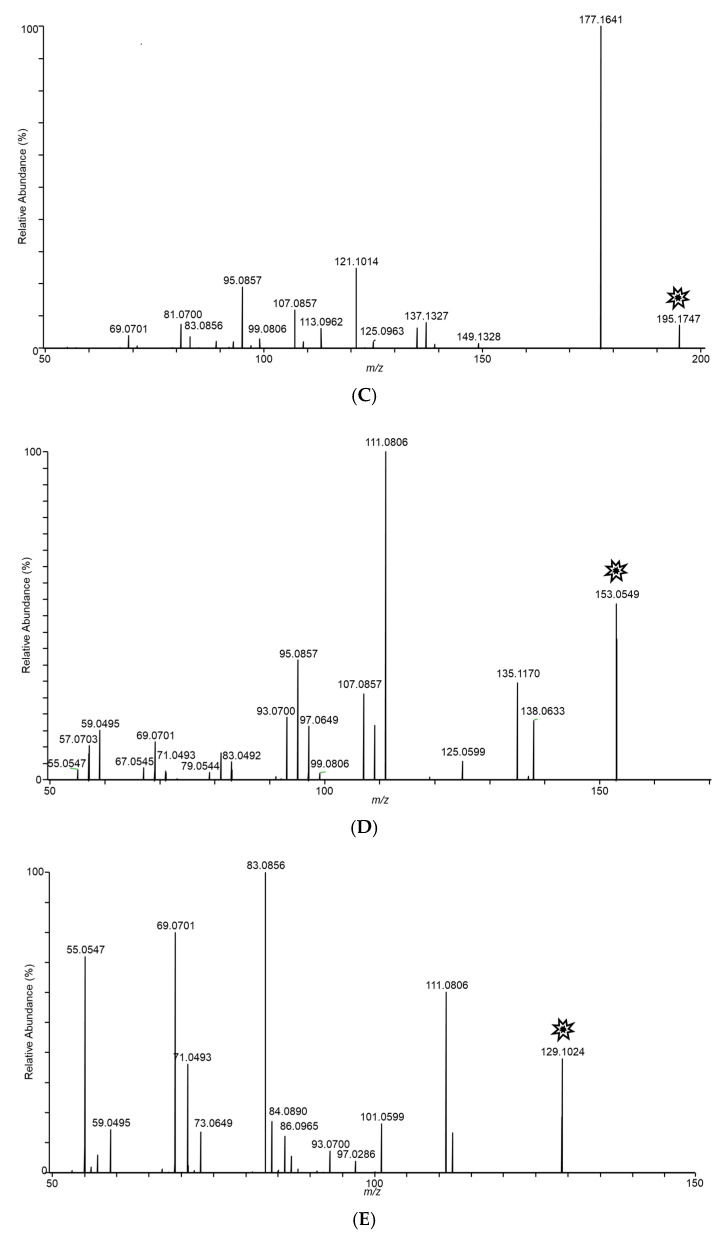

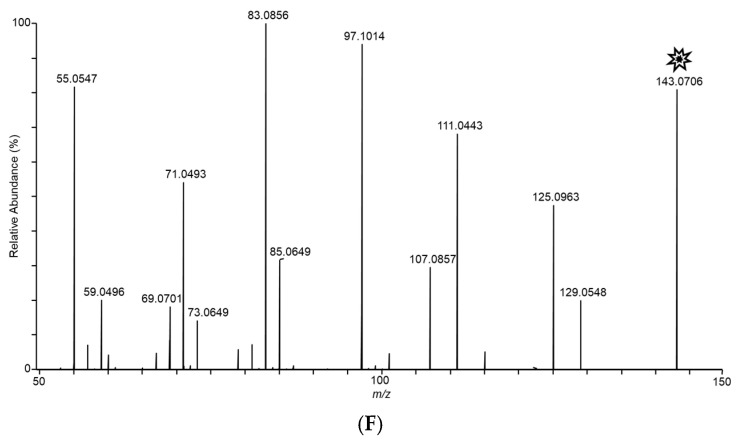
Tandem CID spectra for *m/z* (**A**) 165 (from *F. scirpi*), (**B**) 167 (from *F. scirpi*), (**C**) 195 (from *F. verticillioides*), (**D**) 153 (from *F. verticillioides*), (**E**) 129 (from *F. graminearum*), and (**F**) 143 (from *F. graminearum*) ions. [M + H]^+^ target ions for the CID experiments are labelled with a “
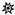
” icon. Spectra represent averaging of the 3 min acquisition period for each sample.

In an effort to structurally characterize the distinctive ions observed for the *Fusarium* isolates, collision-induced dissociation (CID) experiments were conducted to provide structurally informative fragments for the observed ions. Figure 5 shows CID spectra for *m/z* 165, 167, 153, 195, 129, and 143 ions. Despite the presence of multiple fragments in several of the spectra, identities of the observed ions were not conclusively determined.

**Figure 6:**
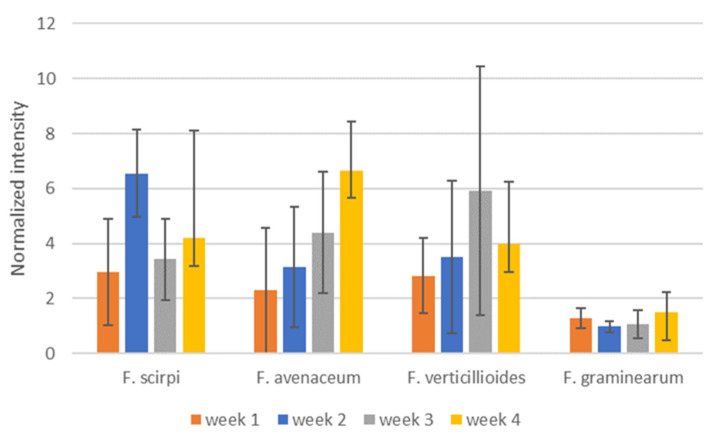
Intensities of selected, potentially diagnostic, ions from weeks 1–4 of sampling for *F scirpi* (*m/z* 167), *F. avenaceum* (*m/z* 83), *F. verticillioides* (*m/z* 195) and *F. graminearum* (*m/z* 129) isolates. Intensities are normalized relative to the diagnostic ion for cracked maize (*m/z* 86). Intensities shown are averages of measurements conducted from culture bottles and error bars indicate calculated standard deviations for the measurements.

To determine if time post-inoculation would influence the species specificity of the selected diagnostic ions, we evaluated the selected ions over the course of the 4-week study. Figure 6 shows levels of selected diagnostic ions for the weeks that measurements were made. Production of the selected VOCs does not appear to dramatically change during the development of fungal colonization of the cracked maize substrate. While DART–MS does have some inherent variability in signal level, the degree of variability in the diagnostic ion intensities here appears to be largely based on biological variability between culture bottles. The variability for the *m/z* 129 ion for *F. graminearum* appears to be less than for the other diagnostic ions.

## 4. Discussion

### 4.1. DART–MS as a Real Time Method for VOC Detection

DART–MS response for sampling of fungal headspace is dependent upon production of volatiles and the chemical properties of these volatiles. Positive mode DART ionization is dependent upon on proton transfer from reagent ions to the analyte. In most cases, the occurrence of this proton transfer is a consequence of the proton affinity of the analyte relative to the proton affinity of water [10]. The bias introduced into the DART–MS headspace analysis by requiring a suitable gas-phase basicity is likely to be similar to other techniques that are also dependent upon proton-transfer reactions in ambient laboratory atmosphere. APCI, HESI, PTR, SIFT, and other related techniques can be expected to have similar inherent VOC selectivities [9]. Previous studies utilizing such techniques have identified several VOCs from *Fusarium* isolates based upon comparison to public databases and published reports of microbial VOCs [12]. The distinctive ions observed here do not appear to overlap with previously published *Fusarium* VOCs. In contrast to techniques dependent upon gas-phase proton-transfer reactions, GC–EI–MS based approaches will have different selectivities. Other researchers have explored the complementary potential in using both EI and proton transfer-based techniques [12].

The trapping step needed for many of the GC–MS and proton transfer approaches introduces another level of bias. VOCs need to be readily absorbed and desorbed in order to be effectively sampled [14]. The direct sampling approach utilized here eliminates the bias associated with the trapping step.

The interface described here is a convenient way to stimulate ionization of sampled volatiles without a trapping step. Further, interface with the MS here offered the sensitivity and resolution to readily distinguish several fungal species. DART–MS can be used with a wide variety of MS analyzers. The choice of analyzer offers a wide selection of cost, portability, sensitivity, mass accuracy, and mass resolution. In addition, interface to tandem mass spectrometry instruments would provide valuable capabilities for obtaining structural information regarding sampled volatiles. The DART–MS technique does not incorporate the chromatographic separation utilized in traditional SPME–GC–MS-based approaches [12]. Recent developments in MS design incorporating ion mobility separation in the MS analysis offers the potential to contribute another dimension in differentiating sampled VOCs. The added dimension from ion mobility could be especially useful in resolving isobaric VOCs that are not easily distinguished by tandem MS behavior [12]. There are advantages to utilization of a dynamic method for VOC characterization, including rapid acquisition of information and convenient observation of changes in VOCs over the course of a short-term biological study. APCI [16], PTR [10], and DART–MS [24] have been shown to be well suited for dynamic VOC determination.

### 4.2. All Fusarium Species May Produce a Variety of VOCs, but VOC Ion Profiles Are Species-Specific

While we did detect species-specific VOC ions, which potentially enable discrimination of *Fusarium* species, it is likely that entire profiles of common volatile ions will provide useful diagnostic information. For example, while several of the VOCs produced by *F. graminearum*, *F. verticillioides*, and *F. scirpi* are the same, the ratios that the common volatiles are produced at differ widely. While the detected VOC levels for the *F. virguliforme* isolate and the *A. flavus* isolate did not readily lead to identification from diagnostic ions, it is possible that the VOC ions detected with high intensity could lead to characterization based on the profile instead of the selected diagnostic ions. Fingerprinting by unique ratios of a suite of common volatiles will require statistical treatment of spectra beyond simple visual inspection of data. A number of studies have shown the utility of statistical methods for using levels of a large number of VOCs for the determination of fungal species [10,11]. While distinctive ions were observed in the work described here, it is likely that utility of the technique will be dependent upon development of statistical tools to facilitate fingerprint analysis to better discriminate between species based upon detection of a broader range of detected VOCs from a wide range of *Fusarium* species.

### 4.3. Rapid Characterization of Fusarium Species Based upon Detected VOCs

Recent decades have seen the classification of *Fusarium* species aided by DNA-based phylogeny. Recently, other approaches have been adopted for rapid species identification, including MALDI–MS protein fingerprinting [25] and SPME–GC–MS VOC fingerprinting. VOC profiling has been used both for species determination and toxicogenic potential. The results here suggest the possibility for use of MS for dynamic determination of species contaminants. Improvements in the MS libraries of VOCs accessible to proton transfer reaction ionization sources will likely aid the utilization of DART–MS based fingerprinting of fungal species. Presently, much of the content of MS libraries is focused on the compounds addressed by EI–MS instrumentation [26].

One possibility not explored here is the dependence of the fungi on the specific conditions utilized in this study. During the conduction of the experiments, care was taken to provide a consistent growth environment for fungal colony development. It is possible that some of the observed VOCs were produced in response to specific conditions prompting fungal growth (inoculation technique, temperature, light, composition of the growth substrate, ventilation of the culture bottle headspace, moisture, etc.). Further studies are required to determine the utility of diagnostic ions from VOCs produced during fungal development under various growth conditions. Results of such studies will do much to indicate potential breadth of future applications of the technique.

## 5. Conclusions

The DART–MS interface described here provides a convenient method for sampling VOCs without sample preparation. Potential exists for rapid, real-time interrogation of a variety of environments, due to the flexible nature of the DART ionization source geometry. The observed VOCs could readily be associated with the fungal species producing them in the headspace above developing fungal colonies.

## Figures and Tables

**Table 1:** Culture isolates utilized in this study.

Species	*Fusarium* Species Complex	NRRL Number
*Fusarium scirpi*	*F. incarnatum–equiseti*	66328
*Fusarium avenaceum*	*F. tricinctum*	4939
*Fusarium virguliforme*	*F. solani*	31041
*Fusarium verticillioides*	*F. fujikuroi*	(M3125) 20956
*Fusarium graminearum*	*F. ambucinum*	31084
*Aspergillus flavus*	–	26466

**Table 2:** Diagnostic ions selected for fungal species. Empirical formulae for diagnostic ions are based upon most probable elemental composition calculated for the high-resolution masses with Thermo Compound Discover.

Species	Diagnostic Ion #1	Diagnostic Ion #2
*Fusarium scirpi*	167.10631 (C_10_H_14_O_2_)	221.19533 (C_15_H_24_O)
*Fusarium avenaceum*	83.08611 (C_6_H_10_)	–
*Fusarium virguliforme*	–	–
*Fusarium verticillioides*	195.06412()	153.12706 (C_10_H_16_O)
*Fusarium graminearum*	129.05443 (C_6_H_8_O_3_)	143.07032 (C_7_H_10_O_3_)
*Aspergillus flavus*	251.13402 (C_10_H_21_O_6_N)	283.16080 (C_11_H_25_O_7_N)

## Data Availability

Not applicable.
